# Supplementary material for: Reporter-ChIP-nexus reveals strong contribution of the Drosophila initiator sequence to RNA polymerase pausing
Source: eLife. 2019 Apr 25;8:e41461. doi: 10.7554/eLife.41461 (PMC6483594; doi:10.7554/eLife.41461)
Supplement: Supplementary file 1. [file elife-41461-supp1.docx]

# Supplementary Information for Shao *et al*. manuscript 2018

This document contains supplementary information and tables, please refer to the main article file for figure and table legends.

## Tables

|  | TATA | Inr | MTE | DPE | PB |
| --- | --- | --- | --- | --- | --- |
| Sequence | STATAWAWR | TCAKTY | CSARCSSA | KCGGTTSK | KCGRWCG |
| Window | -40 to -20 bp | -10 to 10 bp | 20 to 50 bp | 20 to 50 bp | 20 to 50 bp |
| ***Act5C*** | ✓ | ✓ (non-G) | ✓ |  |  |
| ***Pino*** | ✓ | ✓(non-G) |  | ✓ | ✓ |
| ***pepck*** | ✓ | ✓ (G) |  |  |  |
| ***pxb*** |  | ✓ (G) |  |  | ✓ |
| ***comm2*** |  | ✓ (G) |  | ✓ | ✓ |
| ***CG12730*** |  | ✓ (G) |  |  |  |
| ***pk*** |  | ✓ (G) | ✓ |  |  |
| ***dve*** |  | ✓ (G) |  |  |  |
| **SCP** | ✓ | ✓ (G) | ✓ | ✓ | ✓ |

**Table S1. Summary of the core promoter elements present in each analyzed promoter**

| Plasmid construct | Correlation between replicates on the plasmid |
| --- | --- |
| *Act5C* | 0.83 |
| *Act5C_pk_down* | 0.74 |
| *pepck* | 0.94 |
| *pepck_pk_down* | 0.97 |
| *pepck_dve_down* | 0.91 |
| *pk* | 0.91 |
| *TATA-pk* | 0.9 |
| *Act5C_up_pk* | 0.89 |
| *pk_Inr_GtoT* | 0.85 |
| *dve* | 0.97 |
| *TATA-dve* | 0.98 |
| *Act5C_up_dve* | 0.96 |
| *Act5C_up_dve* | 0.96 |
| *Act5C_dve_down* | 0.95 |
| *dve_Act5C_Inr* | 0.93 |
| *dve_Inr_GtoT* | 0.97 |
| *dve_Inr_GtoA* | 0.97 |
| *comm2* | 0.95 |
| *TATA-comm2* | 0.79 |
| *Act5C_up_comm2* | 0.88 |
| SCP | 0.94 |
| *SCP_Inr_GtoT* | 0.87 |

**Table S2. Correlation of Pol II profile on the plasmid between replicates.**

| Promoter | Correlation between plasmid and endogenous |
| --- | --- |
| *Act5C* | 0.83 |
| *Pino* | 0.89 |
| *pepck* | 0.89 |
| *pxb* | 0.87 |
| *comm2* | 0.90 |
| *CG12730* | 0.77 |
| *pk* | 0.90 |
| *dve* | 0.84 |

**Table S3. Correlation between Pol II profile on the plasmid and that at the endogenous locus.**

|  | Pval  Mutually exclusive model | Pval  Non mutually exclusive model |
| --- | --- | --- |
| TATA vs. pausing | 0.01670098 | 4.35E-06 |
| TATA vs. Inr-non-G | 0.65675839 | 0.00084584 |
| TATA vs. Inr-G | 1.58E-08 | 3.75E-26 |
| TATA vs. TATA+pausing | 0.99261039 | 0.65603493 |
| TATA vs. TATA+Inr-non-G | 0.48664501 | 0.0001794 |
| TATA vs. pausing+Inr-non-G | 0.73503479 | 0.00112334 |
| TATA vs. TATA+pausing+Inr-non-G | 0.2259147 | 0.00104381 |
| TATA vs. TATA+Inr-G | 1.94E-05 | 1.17E-07 |
| TATA vs. pausing+Inr-G | 3.21E-11 | 1.14E-26 |
| TATA vs. TATA+pausing+Inr-G | 9.91E-05 | 0.0001948 |
| pausing vs. Inr-non-G | 1.27E-08 | 1.64E-47 |
| pausing vs. Inr-G | 1.21E-25 | 7.42E-35 |
| pausing vs. TATA+pausing | 0.011069 | 9.07E-05 |
| pausing vs. TATA+Inr-non-G | 0.00017977 | 1.08E-13 |
| pausing vs. pausing+Inr-non-G | 1.09E-07 | 1.62E-28 |
| pausing vs. TATA+pausing+Inr-non-G | 3.38E-06 | 9.55E-09 |
| pausing vs. TATA+Inr-G | 2.15E-06 | 0.00017279 |
| pausing vs. pausing+Inr-G | 1.82E-43 | 1.22E-31 |
| pausing vs. TATA+pausing+Inr-G | 5.30E-05 | 0.0189791 |
| Inr-non-G vs. Inr-G | 8.05E-49 | 1.82E-125 |
| Inr-non-G vs. TATA+pausing | 0.68518948 | 0.05768504 |
| Inr-non-G vs. TATA+Inr-non-G | 0.09065448 | 0.00737663 |
| Inr-non-G vs. pausing+Inr-non-G | 0.80864529 | 0.68343018 |
| Inr-non-G vs. TATA+pausing+Inr-non-G | 0.00363388 | 0.01133382 |
| Inr-non-G vs. TATA+Inr-G | 1.56E-10 | 1.54E-18 |
| Inr-non-G vs. pausing+Inr-G | 5.42E-72 | 4.40E-105 |
| Inr-non-G vs. TATA+pausing Inr-G | 8.84E-09 | 2.00E-09 |
| Inr-G vs. TATA+pausing | 5.61E-09 | 7.03E-17 |
| Inr-G vs. TATA+Inr-non-G | 6.90E-12 | 1.01E-27 |
| Inr-G vs. pausing+Inr-non-G | 2.34E-43 | 4.59E-76 |
| Inr-G vs. TATA+pausing+Inr-non-G | 7.00E-14 | 2.51E-16 |
| Inr-G vs. TATA+Inr-G | 0.48260069 | 0.7273453 |
| Inr-G vs. pausing+Inr-G | 0.00160238 | 0.12790065 |
| Inr-G vs. TATA+pausing+Inr-G | 0.92194461 | 0.5788178 |
| TATA+pausing vs. TATA+Inr-non-G | 0.35678806 | 0.00247046 |
| TATA+pausing vs. pausing+Inr-non-G | 0.80458351 | 0.04014878 |
| TATA+pausing vs. TATA+pausing+Inr-non-G | 0.11339781 | 0.00414273 |
| TATA+pausing vs. TATA+Inr-G | 6.89E-06 | 2.67E-07 |
| TATA+pausing vs. pausing+Inr-G | 1.45E-11 | 4.60E-18 |
| TATA+pausing vs. TATA+pausing+Inr-G | 1.66E-05 | 0.00010359 |
| TATA+Inr-non-G vs. pausing+Inr-non-G | 0.15534402 | 0.03006961 |
| TATA+Inr-non-G vs. TATA+pausing+Inr-non-G | 0.39747573 | 0.6177787 |
| TATA+Inr-non-G vs. TATA+Inr-G | 7.30E-07 | 5.85E-13 |
| TATA+Inr-non-G vs. pausing+Inr-G | 2.77E-15 | 1.91E-28 |
| TATA+Inr-non-G vs. TATA+pausing+Inr-G | 3.12E-06 | 1.46E-08 |
| pausing+Inr-non-G vs. TATA+pausing+Inr-non-G | 0.01547637 | 0.03247275 |
| pausing+Inr-non-G vs. TATA+Inr-G | 1.18E-10 | 4.42E-17 |
| pausing+Inr-non-G vs. pausing+Inr-G | 2.63E-63 | 1.86E-70 |
| pausing+Inr-non-G vs. TATA+pausing+Inr-G | 1.18E-08 | 3.82E-09 |
| TATA+pausing+Inr-non-G vs. TATA+Inr-G | 5.77E-08 | 5.15E-10 |
| TATA+pausing+Inr-non-G vs. pausing+Inr-G | 2.90E-17 | 3.87E-17 |
| TATA+pausing+Inr-non-G vs. TATA+pausing+Inr-G | 1.55E-07 | 1.55E-07 |
| TATA+Inr-G vs. pausing+Inr-G | 0.59982049 | 0.33018055 |
| TATA+Inr-G vs. TATA+pausing+Inr-G | 0.69700921 | 0.80950828 |
| pausing+Inr-G vs. TATA+pausing+Inr-G | 0.28862925 | 0.31885584 |

**Table S4. Wilcoxon rank test results between different combinations of core promoter elements.**

| Name | Identity | Modification | Sequence |
| --- | --- | --- | --- |
| Nex_adapter_UBamHI | Adaptor: universal | 5' phosphate | /5Phos/GATCGGAAGAGCACACGTCTGGATCCACGACGCTCTTCC |
| Nex_adapter_BN5BamHI_CTGA | Adaptor: barcoded | 5' phosphate | /5Phos/TCAGNNNNNAGATCGGAAGAGCGTCGTGGATCCAGACGTGTGCTCTTCCGATCT |
| Nex_adapter_BN5BamHI_TGAC | Adaptor: barcoded | 5' phosphate | /5Phos/GTCANNNNNAGATCGGAAGAGCGTCGTGGATCCAGACGTGTGCTCTTCCGATCT |
| Nex_adapter_BN5BamHI_GACT | Adaptor: barcoded | 5' phosphate | /5Phos/AGTCNNNNNAGATCGGAAGAGCGTCGTGGATCCAGACGTGTGCTCTTCCGATCT |
| Nex_adapter_BN5BamHI_ACTG | Adaptor: barcoded | 5' phosphate | /5Phos/CAGTNNNNNAGATCGGAAGAGCGTCGTGGATCCAGACGTGTGCTCTTCCGATCT |
| Nex_cut_BamHI | Oligo for digestion | None | GAAGAGCGTCGTGGATCCAGACGTG |
| Nex_primer_U | Primer: universal | 3' phosphoro-thioate bond | AATGATACGGCGACCACCGAGATCTACACTCTTTCCCTACACGACGCTCTTCCGATC*T |
| Nex_primer_B01 | Primer: barcoded | 3' phosphoro-thioate bond | CAAGCAGAAGACGGCATACGAGATCGTGATGTGACTGGAGTTCAGACGTGTGCTCTTCCGATC*T |
| Nex_primer_B02 | Primer: barcoded | 3' phosphoro-thioate bond | CAAGCAGAAGACGGCATACGAGATACATCGGTGACTGGAGTTCAGACGTGTGCTCTTCCGATC*T |
| Nex_primer_B03 | Primer: barcoded | 3' phosphoro-thioate bond | CAAGCAGAAGACGGCATACGAGATGCCTAAGTGACTGGAGTTCAGACGTGTGCTCTTCCGATC*T |
| Nex_primer_B04 | Primer: barcoded | 3' phosphoro-thioate bond | CAAGCAGAAGACGGCATACGAGATTGGTCAGTGACTGGAGTTCAGACGTGTGCTCTTCCGATC*T |
| Nex_primer_B05 | Primer: barcoded | 3' phosphoro-thioate bond | CAAGCAGAAGACGGCATACGAGATCACTGTGTGACTGGAGTTCAGACGTGTGCTCTTCCGATC*T |
| Nex_primer_B06 | Primer: barcoded | 3' phosphoro-thioate bond | CAAGCAGAAGACGGCATACGAGATATTGGCGTGACTGGAGTTCAGACGTGTGCTCTTCCGATC*T |
| Nex_primer_B07 | Primer: barcoded | 3' phosphoro-thioate bond | CAAGCAGAAGACGGCATACGAGATGATCTGGTGACTGGAGTTCAGACGTGTGCTCTTCCGATC*T |
| Nex_primer_B08 | Primer: barcoded | 3' phosphoro-thioate bond | CAAGCAGAAGACGGCATACGAGATTCAAGTGTGACTGGAGTTCAGACGTGTGCTCTTCCGATC*T |
| Nex_primer_B09 | Primer: barcoded | 3' phosphoro-thioate bond | CAAGCAGAAGACGGCATACGAGATCTGATCGTGACTGGAGTTCAGACGTGTGCTCTTCCGATC*T |
| Nex_primer_B10 | Primer: barcoded | 3' phosphoro-thioate bond | CAAGCAGAAGACGGCATACGAGATAAGCTAGTGACTGGAGTTCAGACGTGTGCTCTTCCGATC*T |
| Nex_primer_B11 | Primer: barcoded | 3' phosphoro-thioate bond | CAAGCAGAAGACGGCATACGAGATGTAGCCGTGACTGGAGTTCAGACGTGTGCTCTTCCGATC*T |
| Nex_primer_B12 | Primer: barcoded | 3' phosphoro-thioate bond | CAAGCAGAAGACGGCATACGAGATTACAAGGTGACTGGAGTTCAGACGTGTGCTCTTCCGATC*T |
| Nex_primer_B13 | Primer: barcoded | 3' phosphoro-thioate bond | CAAGCAGAAGACGGCATACGAGATTTGACTGTGACTGGAGTTCAGACGTGTGCTCTTCCGATC*T |
| Nex_primer_B14 | Primer: barcoded | 3' phosphoro-thioate bond | CAAGCAGAAGACGGCATACGAGATGGAACTGTGACTGGAGTTCAGACGTGTGCTCTTCCGATC*T |
| Nex_primer_B15 | Primer: barcoded | 3' phosphoro-thioate bond | CAAGCAGAAGACGGCATACGAGATTGACATGTGACTGGAGTTCAGACGTGTGCTCTTCCGATC*T |
| Nex_primer_B16 | Primer: barcoded | 3' phosphoro-thioate bond | CAAGCAGAAGACGGCATACGAGATGGACGGGTGACTGGAGTTCAGACGTGTGCTCTTCCGATC*T |
| Nex_primer_B18 | Primer: barcoded | 3' phosphoro-thioate bond | CAAGCAGAAGACGGCATACGAGATGCGGACGTGACTGGAGTTCAGACGTGTGCTCTTCCGATC*T |
| Nex_primer_B19 | Primer: barcoded | 3' phosphoro-thioate bond | CAAGCAGAAGACGGCATACGAGATTTTCACGTGACTGGAGTTCAGACGTGTGCTCTTCCGATC*T |
| Nex_primer_B20 | Primer: barcoded | 3' phosphoro-thioate bond | CAAGCAGAAGACGGCATACGAGATGGCCACGTGACTGGAGTTCAGACGTGTGCTCTTCCGATC*T |

**Table S5. Summary of ChIP-nexus oligos**

| Name | Identity | Modification | Sequence |
| --- | --- | --- | --- |
| GFP_reversetx_primer | Reverse transcription primer | 5' phosphate | /5Phos/TCAGAGATCGGAAGAGCGTCGTGGATCCAGATGAACTTCAGGGTCAGCTT |
| Reversetx_BamHI_cutting | Oligo for digestion | None | AAGTTCATCTGGATCCACGACGCTC |
| Reversetx_universal_primer | Primer: universal | None | AATGATACGGCGACCACCGAGATCTACACTCTTTCCCTACACGACGCTCTTCCGATCT |
| Reversetx_barcode_primer_#1 | Primer: barcoded | None | CAAGCAGAAGACGGCATACGAGATCGTGATGTGACTGGAGTTCAGACGTGTGCTCTTCCGATCTACGCTGAACTTGTGGCCGTTTA |
| Reversetx_barcode_primer_#2 | Primer: barcoded | None | CAAGCAGAAGACGGCATACGAGATACATCGTGTGACTGGAGTTCAGACGTGTGCTCTTCCGATCTACGCTGAACTTGTGGCCGTTTA |
| Reversetx_barcode_primer_#3 | Primer: barcoded | None | CAAGCAGAAGACGGCATACGAGATGCCTAAGTGACTGGAGTTCAGACGTGTGCTCTTCCGATCTACGCTGAACTTGTGGCCGTTTA |
| Reversetx_barcode_primer_#4 | Primer: barcoded | None | CAAGCAGAAGACGGCATACGAGATTGGTCAGTGACTGGAGTTCAGACGTGTGCTCTTCCGATCTACGCTGAACTTGTGGCCGTTTA |

**Table S6. Summary of gene-specific 5’ RNA sequencing oligos**

| Name | Source and description |
| --- | --- |
| pAWG | *Drosophila* Gateway vector collection |
| pAWG_UAS | 6x UAS was interted after removing *Act5C* core promoter and gateway cassette at pAWG_UAS, an EcoRV cutting site was introduced between UAS and eGFP for promoter insertion |
| pAWG_UAS_dps_Act5C | *dps* *Act5C* promoter sequence was inserted into pAWG_UAS |
| pAWG_UAS_dps_Act5C_2kb | *dps* *Act5C* 2kb region was inserted into pAWG_UAS |
| pAWG_UAS_dps_Act5C_dve_down | *dps* *Act5C* downstream promoter sequence was replaced with *dps dve* downstream promoter sequence and inserted into pAWG_UAS. Fusion site: 8 bp after the TSS |
| pAWG_UAS_dps_Act5C_pk_down | *dps* *Act5C* downstream promoter sequence was replaced with *dps pk* downstream promoter sequence and inserted into pAWG_UAS. Fusion site: 8 bp after the TSS |
| pAWG_UAS_dps_Act5C_up_dve | *dps* *dve* upstream promoter sequence was replaced with *dps Act5C* upstream promoter sequence and inserted into pAWG_UAS. Fusion site: 7 bp before the TSS |
| pAWG_UAS_dps_Act5C_up_pk | *dps* *pk* upstream promoter sequence was replaced with *dps Act5C* upstream promoter sequence and inserted into pAWG_UAS. Fusion site: 7 bp before the TSS |
| pAWG_UAS_dps_Act5C_up_comm2 | *dps* *comm2* upstream promoter sequence was replaced with dps Act5C upstream promoter sequence and inserted into pAWG_UAS. Fusion site: 7 bp before the TSS |
| pAWG_UAS_dps_CG12730 | *dps* *CG12730* promoter sequence was inserted into pAWG_UAS |
| pAWG_UAS_dps_comm2 | *dps* *comm2* promoter sequence was inserted into pAWG_UAS |
| pAWG_UAS_dps_comm2_tata_insert | A 7 bp region 31 bp upstream of *dps comm2* TSS was replaced with TATAAAA at  pAWG_UAS_dps_comm2 |
| pAWG_UAS_dps_dve | *dps* *dve* promoter sequence was inserted into pAWG_UAS |
| pAWG_UAS_dps_dve_tata_insert | A 7 bp region 31 bp upstream of *dps dve* TSS was replaced with TATAAAA at  pAWG_UAS_dps_dve |
| pAWG_UAS_dps_dve_Act5C_inr | *dps* *dve* Inr sequence (16 bp around the TSS) was replaced with *dps Act5C* Inr sequence at pAWG_UAS_dps_dve |
| pAWG_UAS_dps_dve_inr_GtoA | *dps* *dve* Inr sequence at position +2 was mutated to A at pAWG_UAS_dps_dve |
| pAWG_UAS_dps_dve_inr_GtoT | *dps* *dve* Inr sequence at position +2 was mutated to T at pAWG_UAS_dps_dve |
| pAWG_UAS_dps_pepck | *dps* *pepck* promoter sequence was inserted into pAWG_UAS |
| pAWG_UAS_dps_pepck_dve_down | *dps* *pepck* downstream promoter sequence was replaced with *dps dve* downstream promoter sequence and inserted into pAWG_UAS. Fusion site: 8 bp after the TSS |
| pAWG_UAS_dps_pepck_pk_down | *dps* *pepck* downstream promoter sequence was replaced with *dps pk* downstream promoter sequence and inserted into pAWG_UAS. Fusion site: 8 bp after the TSS |
| pAWG_UAS_dps_Pino | *dps* *Pino* promoter sequence was inserted into pAWG_UAS |
| pAWG_UAS_dps_pk | *dps* *pk* promoter sequence was inserted into pAWG_UAS |
| pAWG_UAS_dps_pk_tata_insert | A 7 bp region 31 bp upstream of dps pk TSS was replaced with TATAAAA at  pAWG_UAS_dps_pk |
| pAWG_UAS_dps_pk_inr_GtoT | *dps* *pk* Inr sequence at position +2 was mutated to T at pAWG_UAS_dps_pk |
| pAWG_UAS_dps_pxb | *dps* *pxb* promoter sequence was inserted into pAWG_UAS |
| pAWG_UAS_dps_RpL13A | *dps* *RPL13A* promoter sequence was inserted into pAWG_UAS |
| pAWG_UAS_dps_RpL13A_2kb | *dps* *RPL13A* 2kb region was inserted into pAWG_UAS |
| pAWG_UAS_SCP | Super core promoter was inserted into pAWG_UAS |
| pAWG_UAS_SCP_inr_GtoT | SCP Inr sequence at position +2 was mutated to T at pAWG_UAS_SCP |
| SCP1-pUC119 plasmid | Addgene #21846 |

**Table S7. Summary of all plasmids used in this study**

| Name | Sequence |
| --- | --- |
| pAWG_UAS | GCGCCCAATACGCAAACCGCCTCTCCCCGCGCGTTGGCCGATTCATTAATGCAGGCAACTCGTGAAAGGTAGGCGGATCAGCGGCCGCAGCATGCAATTCTATATTCTAAAAACACAAATGATACTTCTAAAAAAAAATCATGAATGGCATCAACTCTGAATCAAATCTTTGCAGATGCACCTACTTCTCATTTCCACTGTCACATCATTTTTCCAGATCTCGCTGCCTGTTATGTGGCCCACAAACCAAGACACGTTTTATGGCCATTAAAGCTGGCTGATCGTCGCCAAACACCAAATACATAATGAATATGTACACATTCGAGAAAGAAGCGATCAAAGAAGCGTCTTCGGGCGGAGTAGGAGAATGCGGAGGAGAAGGAGAACGAGCTGATCTAGTATCTCTCCACAATCCAATGCCAACTGACCAACTGGCCATATTCGGAGCAATTTGAAGCCAATTTCCATCGCCTGGCGATCGCTCCATTCTTGGCTATATGTTTTTCACCGTTACCCGGGGCCATTTTCAAAGACTCGTCGGCAAGATAAGATTGTGTCACTCGCTGTCTCTCTTCATTTGTCGAAGAATGCTGAGGAATTTCGCGATGACGTCGGCGAGTATTTTGAAGAATGAGAATAATTTGTATTTATACGAAAATCAGTTAGTGGAATTTTCTACAAAAACATGTTATCTATAGATAATTTTGTTGCAAAATATGTTGACTATGACAAAGATTGTATGTATATACCTTTAATGTATTCTCATTTTCTTATGTATTTATAATGGCAATGATGATACTGATGATATTTTAAGATGATGCCAGACCAAAAGGCTTGAATTTCTGCGTCTTTTGCCGAACGCAGTGCATGTGCAATTGTTGTTTTTTGGAATATTCAATTTTCGGACTGTCCGCTTTGATTTCAGTTTCTTGGCTTATTCAAAAAGCAAAGTAAAGCCAAAAAAGCGAGATGGCAATACCAAATGCGGCAAAACGGTAGTGGAAGGAAAGGGGTGCGGGGCAGCGGAAGGAAGGGTGGGGCGGGGCGTGGCGGGGTCTGTGGCTGGGCGCGACGTCACCGACGTTGGAGCCACTCCTTTGACCATGTGTGCGTGTGTGTATTATTCGTGTCTCGCCACTCGCCGGTTGTTTTTTTCTTTTTATGCTGCGCTCTCTCTAGCGCCATCTCGCTTACGCATGCTCAACGCACCGCATGTTGCCGTTTCCTTTTATGCGTCATTTTGGCTCGAAATAGGCAATTATTTAAACAAAGATTAGTCAACGAAAACGCTAAAATAAATAAGTCTACAATATGGTTACTTATTGCCATGTGTGTGCAGCCAACGATAGCAACAAAAGCAACAACACAGGTGGCTTTCCCTCTTTCACTTTTTGTTTGCAAGCCGCGTGCGAGCAAGACGGCACGACCGGCAAACGCAATTACGCTGACAAAGAGCAGACGAAGTTTTGGCGAAAAACATCAAGGCGCCTGATACGAATGCATTTGCAATAACAATTGCGATATTTAATATTGTTTATGAAGCTGTTTGACTTCAAAACACACAAAAAAAAAAATAAAACAAATTATTTGAAAGAGAATTAGGAATCGGACGCTTATCGTTAGGGTAACAACAAGAAATGCTTACTGAGTCACAGCCTCTGGAAAACTGCCGCAAGCCAGAGAGAGAGAGAAAAAGAGGGAGAGCAGCTTAGACCGCATGTGCTTGTGTGTGAGGCGTCTCTCTCTTCGTCTCTGTTGCGCAAACGCATAGACTGCACTGAGAAAATCGATTACCTATTTTTTATGAATGAATATTTGCACTATTACTATTCAAAACTATTAAGATAGCAATCACATTCAATAGCCAAATACTATACCACCTGAGCGATGCAACGAAATGATCAATTTGAGCAAAAATGCTGCATATTTAGGACGGCATCATTATAGAAATGCTTCTTGCTGTGTACTTTTCTCTCGTCTGGCAGCTGTTTCGCCGTTATTGTTAAAACCGGCTTAAGTTAGGTGTGTTTTCTACGACTAGTGAATGCCCTACTAGAAGATGTGTGTTGCACAAAATGTCCCTGGAATAACCAATTTGAAGTGCAGATAGCAGTAAACGTAAGCTAATATGAATATTATTTAACTGTAATGTTTTAATATCGCTGGACATTACTAATAAACCCACTATAAACACATGTACATATGTATGTTTTGGCATACAATGAGTAGTTGGGGAAAAAATGTGTAAAAGCACCGTGACCATCACAGCATAAAGATAACCAGCTGAAGTATCGAATATGAGTAACCCCCAAATTGAATCACATGCCGCAACTGATAGGACCCATGGAAGTACACTCTTCATGGCGATATACAAGACACACACAAGCACGAACACCCAGTTGCGGAGGAAATTCTCCGTAAATGAAAACCCAATCGGCGAACAATTCATACCCATATATGGTAAAAGTTTTGAACGCGACTTGAGAGCGGAGAGCATTGCGGCTGATAAGGTTTTAGCATCAAGCTTAGGCCTCCAAGGCGGAGTACTGTCCTCCGGGCTGGCGGAGTACTGTCCTCCGGCAAGGCGGAGTACTGTCCTCCGGGCTGGCGGAGTACTGTCCTCCGGCAAGGCGGAGTACTGTCCTCCGGGCTGGCGGAGTACTGTCCTCCGGCAAGGCGGAGTACTGTCCTCCGGGCTGGCGGAGTACTGTCCTCCGGCAAGGCGGAGTACTGTCCTCCGGGCTGGCGGAGTACTGTCCTCCGGCAAGGCGGAGTACTGTCCTCCGGGCTGGCGGAGTACTGTCCTCCGGCAAGGCGGAGTACTGTCCTCCGGGCTGGCGGAGTACTGTCCTCCGGCAAGGGTCGAGTCGATAGCCGAGATATCCGCCACCATGGTGAGCAAGGGCGAGGAGCTGTTCACCGGGGTGGTGCCCATCCTGGTCGAGCTGGACGGCGACGTAAACGGCCACAAGTTCAGCGTGTCCGGCGAGGGCGAGGGCGATGCCACCTACGGCAAGCTGACCCTGAAGTTCATCTGCACCACCGGCAAGCTGCCCGTGCCCTGGCCCACCCTCGTGACCACCCTGACCTACGGCGTGCAGTGCTTCAGCCGCTACCCCGACCACATGAAGCAGCACGACTTCTTCAAGTCCGCCATGCCCGAAGGCTACGTCCAGGAGCGCACCATCTTCTTCAAGGACGACGGCAACTACAAGACCCGCGCCGAGGTGAAGTTCGAGGGCGACACCCTGGTGAACCGCATCGAGCTGAAGGGCATCGACTTCAAGGAGGACGGCAACATCCTGGGGCACAAGCTGGAGTACAACTACAACAGCCACAACGTCTATATCATGGCCGACAAGCAGAAGAACGGCATCAAGGTGAACTTCAAGATCCGCCACAACATCGAGGACGGCAGCGTGCAGCTCGCCGACCACTACCAGCAGAACACCCCCATCGGCGACGGCCCCGTGCTGCTGCCCGACAACCACTACCTGAGCACCCAGTCCGCCCTGAGCAAAGACCCCAACGAGAAGCGCGATCACATGGTCCTGCTGGAGTTCGTGACCGCCGCCGGGATCACTCTCGGCATGGACGAGCTGTACAAGCACCGGTCCACGTGACGTAAGCTAGCAGGATCTTTGTGAAGGAACCTTACTTCTGTGGTGTGACATAATTGGACAAACTACCTACAGAGATTTAAAGCTCTAAGGTAAATATAAAATTTTTAAGTGTATAATGTGTTAAACTACTGATTCTAATTGTTTGTGTATTTTAGATTCCAACCTATGGAACTGATGAATGGGAGCAGTGGTGGAATGCCTTTAATGAGGAAAACCTGTTTTGCTCAGAAGAAATGCCATCTAGTGATGATGAGGCTACTGCTGACTCTCAACATTCTACTCCTCCAAAAAAGAAGAGAAAGGTAGAAGACCCCAAGGACTTTCCTTCAGAATTGCTAAGTTTTTTGAGTCATGCTGTGTTTAGTAATAGAACTCTTGCTTGCTTTGCTATTTACACCACAAAGGAAAAAGCTGCACTGCTATACAAGAAAATTATGGAAAAATATTCTGTAACCTTTATAAGTAGGCATAACAGTTATAATCATAACATACTGTTTTTTCTTACTCCACACAGGCATAGAGTGTCTGCTATTAATAACTATGCTCAAAAATTGTGTACCTTTAGCTTTTTAATTTGTAAAGGGGTTAATAAGGAATATTTGATGTATAGTGCCTTGACTAGAGATCATAATCAGCCATACCACATTTGTAGAGGTTTTACTTGCTTTAAAAAACCTCCCACACCTCCCCCTGAACCTGAAACATAAAATGAATGCAATTGTTGTTGTTAACTTGTTTATTGCAGCTTATAATGGTTACAAATAAAGCAATAGCATCACAAATTTCACAAATAAAGCATTTTTTTCACTGCATTCTAGTTGTGGTTTGTCCAAACTCATCAATGTATCTTATCATGTCTGGATCCCGTTTAAACTACGCGTAATTCAAACAGGGTTCTGGCGTCGTTCTCGTACTGTTTTCCCCAGGCCAGTGCTTTAGCGTTATTGAAAAAGGAAGAGTATGAGTATTCAACATTTCCGTGTCGCCCTTATTCCCTTTTTTGCGGCATTTTGCCTTCCTGTTTTTGCTCACCCAGAAACGCTGGTGAAAGTAAAAGATGCTGAAGATCAGTTGGGTGCACGAGTGGGTTACATCGAACTGGATCTCAACAGCGGTAAGATCCTTGAGAGTTTTCGCCCCGAAGAACGTTTTCCAATGATGAGCACTTTTAAAGTTCTGCTATGTGGCGCGGTATTATCCCGTATTGACGCCGGGCAAGAGCAACTCGGTCGCCGCATACACTATTCTCAGAATGACTTGGTTGAGTACTCACCAGTCACAGAAAAGCATCTTACGGATGGCATGACAGTAAGAGAATTATGCAGTGCTGCCATAACCATGAGTGATAACACTGCGGCCAACTTACTTCTGACAACGATCGGAGGACCGAAGGAGCTAACCGCTTTTTTGCACAACATGGGGGATCATGTAACTCGCCTTGATCGTTGGGAACCGGAGCTGAATGAAGCCATACCAAACGACGAGCGTGACACCACGATGCCTGTAGCAATGGCAACAACGTTGCGCAAACTATTAACTGGCGAACTACTTACTCTAGCTTCCCGGCAACAATTAATAGACTGGATGGAGGCGGATAAAGTTGCAGGACCACTTCTGCGCTCGGCCCTTCCGGCTGGCTGGTTTATTGCTGATAAATCTGGAGCCGGTGAGCGTGGGTCTCGCGGTATCATTGCAGCACTGGGGCCAGATGGTAAGCCCTCCCGTATCGTAGTTATCTACACGACGGGGAGTCAGGCAACTATGGATGAACGAAATAGACAGATCGCTGAGATAGGTGCCTCACTGATTAAGCATTGGTAACTGTCAGACCAAGTTTACTCATATATACTTTAGATTGATTTAAAACTTCATTTTTAATTTAAAAGGATCTAGGTGAAGATCCTTTTTGATAATCTCATGACCAAAATCCCTTAACGTGAGTTTTCGTTCCACTGAGCGTCAGACCCCGTAGAAAAGATCAAAGGATCTTCTTGAGATCCTTTTTTTCTGCGCGTAATCTGCTGCTTGCAAACAAAAAAACCACCGCTACCAGCGGTGGTTTGTTTGCCGGATCAAGAGCTACCAACTCTTTTTCCGAAGGTAACTGGCTTCAGCAGAGCGCAGATACCAAATACTGTCCTTCTAGTGTAGCCGTAGTTAGGCCACCACTTCAAGAACTCTGTAGCACCGCCTACATACCTCGCTCTGCTAATCCTGTTACCAGTGGCTGCTGCCAGTGGCGATAAGTCGTGTCTTACCGGGTTGGACTCAAGACGATAGTTACCGGATAAGGCGCAGCGGTCGGGCTGAACGGGGGGTTCGTGCACACAGCCCAGCTTGGAGCGAACGACCTACACCGAACTGAGATACCTACAGCGTGAGCTATGAGAAAGCGCCACGCTTCCCGAAGGGAGAAAGGCGGACAGGTATCCGGTAAGCGGCAGGGTCGGAACAGGAGAGCGCACGAGGGAGCTTCCAGGGGGAAACGCCTGGTATCTTTATAGTCCTGTCGGGTTTCGCCACCTCTGACTTGAGCGTCGATTTTTGTGATGCTCGTCAGGGGGGCGGAGCCTATGGAAAAACGCCAGCAACGCGGCCTTTTTACGGTTCCTGGCCTTTTGCTGGCCTTTTGCTCACATGTTCTTTCCTGCGTTATCCCCTGATTCTGTGGATAACCGTATTACCGCCTTTGAGTGAGCTGATACCGCTCGCCGCAGCCGAACGACCGAGCGCAGCGAGTCAGTGAGCGAGGAAGCGGAAGA |
| *dps Act5C* | ATATGGTAAACTTGGCTGCGGAATGGAGAAATGTCGCTGATAAGGAGTTGAGCGCAGCATGAGCGTGAGTGACGTAGCCGGCTGGAAAGCGGGCTTTATAAAACGGCAAGCAACGACCAGTTTTCATATCATTACAGTTTGAGTTCTTGTGCTGTGTGGATATTCCCAACCGACACAACACCGCTCTACAATCAGCCAGCAGTCGTCTACGCAAAAGACACTCAACCGAAAGACTTAA |
| *dps Act5C 2kb* | ACTTTACAATTTGCCAAATTGTTAACAAATTTTTAATGTGAGAGTTCGATTCATACGAAAAACGCAACATTGTGTATGTTAAAATATATGTATGTATGAAAAAAGCTTTACAAAAGCATGTGGATTGAAGGTTATTTTAAATAAATTTCAGGGAATGGAACGATAAGAATTGATTATTGCGTGGTCATGAGTAATTGCCTGAGTCACATGTAAGAATTTGCAGATACGCACTGACCGATGCAAGAGGTTTTTAAAACAAGCCCAAAAACAAAGTGAACGAACCCCTCGCGAACCAATACACAACTACAATACACCATTGAGAGCAGGCACAGCACACACCCATGTTCACTCGCTCACCACACCACACCAGCTCGTCTTGTGAGCGTGGAAATTCTCTTGAAAATCGACTGGCGAACAATTCATACCCATATATGGTAAACTTGGCTGCGGAATGGAGAAATGTCGCTGATAAGGAGTTGAGCGCAGCATGAGCGTGAGTGACGTAGCCGGCTGGAAAGCGGGCTTTATAAAACGGCAAGCAACGACCAGTTTTCATATCATTACAGTTTGAGTTCTTGTGCTGTGTGGATATTCCCAACCGACACAACACCGCTCTCCAATCAGCCAGCAGTCGTCTACGCAAAAGACACTCAACCGAAAGACTTAATTTATATTAATTTAATTAATTTTAAACAAAACAGACCAAATGTAAGTATACTAAAAAGTTCGAAACGATACGATCCAGAAAAGTCCGAGAATCCGTCCCCCCACCGTCCCTTTAAAAAAGCCATCGCAAACCGTGAGACCGAAAAAAAAAAACAGTGCAAATGTTTTTGTATGATAACTACATTTTTTTGTGTGATTGTGTGAAAAGTGTTGGACAAGTTTGACGAAAAATATGCAAATGAAAATGTTATTCTTAACAGAAAAGTTGATGACATTAAAAAAGCAGAGTTTGCTGGAAAATCTCCCCATGGCATATGTCATTCCAAAAGAGGAATTTGACGGGGAAGCGTCTTAGCTGAGAATGCACCCACAGAACTGCTGCAACTACTATTGTTATCATTCAGATTAGTTCTAATCTACCTAGACATGTACATATATATGTATGTACTATCTGTATCTGCAGATGACTGGGCGTGTCACGCGCCATTTTACGATTTCACCCAAGACAGCCAAGAGGGGAAAGAAGTAGACCGAAATCTTTGGCGACAGTTTCGTTTTTGCCATTTGAAATTCAATTCGCGAACCGCTTTCTTTTTCCCACGCCCAAAAATAACTTTCCCTGAAAGCGAGATTCGCAAAATAATTGTTAGACGTTCACTGTGGCCGGCCCACGCAAAGCCCCAAGAAAGATCTGTAGCAGATCATGGTTATAGGTCCCATGCCATAGAACAGACTTGTCGCGCCTGTGACGTAGGCAGATCACAATCCATAAATGTTTGTGGCGTTTGTAGGCGGCCAGAAATAGTAACAGAATCGCGTCATTAAAGTTATTTGGAAAGACTTGATCGGCGGGGCTCTAAAATGCCGGCTTCAATAAATACGTACATGTGCATGCACATATGTATGTGTACAAGTTGCCGAAGTCACTGCCGTTGTAGCTGTGCAAAAATGGAGAAAACCTTCGGCGTCAGTCTCGGGAGCATGCGAGTGTGTTTCTGTGTGTGAGTGTGTGTGTGTGTGAGAGCGAAAGCGAGCGTTTCGCTTCGAAACATGACCATATATGGTCACAAAAGTTGGCTTCGTCGATTCGACTCGTTCGCTCTCTGTCGCACGCATGCGCTGGCAGCCGCTCAAAATGTGCAGGCGAGAGCGAGTCGGCACACACATACACGAGCGCACACCCGAGACAGCTATCGCGTCAAAGCCGACGCTGCCAGCGCCATTGAATCGTTGTTAACTAGCATTGCCGCTGCCACAGCCCGGCCTGTTCTGTTCATTCCTCTTTCGGCGGTCAAACGGTGCGGTTGCATTAACTTTTCGAAATCCCAACCAAGTTGTTGTGCGAACATCAGGTTTTCCCTTTAAACTCGTTTTTTGAAACAAAAAAGGTAAATTAAATAGTGCTGAAAAAAATTAAAATCAAAGTGGCTTAATTGAAAACTACATTTCACTGCACATATTACGCGATTTTTTGGCCAAATGCCTTGTTAGCCGGCGCTGAAAGCGCCTAGAAGTTTCTTCTTTTTTTCAAGTTTAGTGTAACTGTTCCTTTTTGGTTTAAAATGGAAAGAAAATATACGCGTGATTCTAGATTGTTCTGCTAGACCGGCATCTATAAAAATCTCGTTTTTTCTCTTTCTGTGTTCTTTTTCGGCCGCCATTTTACAGCGCCTTTGCCGCATACACACACACACACACACGCGCGCACACAGACAAGCACTCTTTTGCACCGGCATTCGAGTGCTTTGTGTCTTGGCTGTTCTTTCTTTCGGGGTGGGGCCCTTCCCTGCGGCTCAAACTTGCAATTCATCCTGTGCCTCTCGCTCTTTCTCGCGAAACTTTGAATTTAACCGGAACTAATTTTGTTGTCTTTTGTGTATCCTTGCAGCTTACAATATGTGTGACGAAGAAGTTGCTGCTCTCGTTGTCGACAATGGCTCCGGCATGTGCAAGGCCGGTTTCGCTGGAGATGATGCACCCCGCGCCGTGTTCCCATCGATCGTCGGCCGCCCACGTCACCAGGGTGTCATGGTTGGCATGGGACAGAAGGACTCGTATGTGGGTGATGAGGCCCAGAGCAAGCGTGGTATCCTCACTCTGAAGTACCCCATCGAGCACGGAATCGTGACAAACTGGGATGATATGGAGAAGATCTGGCATCACACCTTCTACAACGAGCTGCGTGTTGCCCCCGAGGAGCACCCCGTTCTGCTGACCGAGGCCCCTCTGAACCCCAAGGCTAACCGTGAGAAGATGACTCAGATCATGTTCGAGACCTTCAA |
| *dps Act5C_dve_down* | ATATGGTAAACTTGGCTGCGGAATGGAGAAATGTCGCTGATAAGGAGTTGAGCGCAGCATGAGCGTGAGTGACGTAGCCGGCTGGAAAGCGGGCTTTATAAAACGGCAAGCAACGACCAGTTTTCATATCATTATCAAACATGCTCGAAGAAGGTTTGTTACTCTGCTCTCTCTTGCATCTCGAGTTTTCGGGTTCTCGGCTTCTTGTGTTTTGTGTGTTCCGCGCCGGTTTCCGTTCTAGTATCTGTGTGTGTTTCTGTTTGGCCGTATTTATGCTTGATGGCCAGCAATCGGATTATGT |
| *dps Act5C_pk_down* | ATATGGTAAACTTGGCTGCGGAATGGAGAAATGTCGCTGATAAGGAGTTGAGCGCAGCATGAGCGTGAGTGACGTAGCCGGCTGGAAAGCGGGCTTTATAAAACGGCAAGCAACGACCAGTTTTCATATCATTATCAGACACGATCACGCGCGCGCACTCTCGCCCCACGTTGAAAAGTCCATGCTGCGCATGCGCGAAACGGAGTGAAAGAAACGTTATTGCAACTGCGACTGCCCCCGCGTTTTGGGCTGGCCTTCTTATCAAGT |
| *dps Act5C_up_dve* | ATATGGTAAACTTGGCTGCGGAATGGAGAAATGTCGCTGATAAGGAGTTGAGCGCAGCATGAGCGTGAGTGACGTAGCCGGCTGGAAAGCGGGCTTTATAAAACGGCAAGCAACGACCCGGTTTCAGTATCTTTTCAAACATGCTCGAAGAAGGTTTGTTACTCTGCTCTCTCTTGCATCTCGAGTTTTCGGGTTCTCGGCTTCTTGTGTTTTGTGTGTTCCGCGCCGGTTTCCGTTCTAGTATCTGTGTGTGTTTCTGTTTGGCCGTATTTATGCTTGATGGCCAGCAATCGGATTATGT |
| *dps Act5C_up_pk* | ATATGGTAAACTTGGCTGCGGAATGGAGAAATGTCGCTGATAAGGAGTTGAGCGCAGCATGAGCGTGAGTGACGTAGCCGGCTGGAAAGCGGGCTTTATAAAACGGCAAGCAACGACCCGCCGTCAGTCTGCTTTCAGACACGATCACGCGCGCGCACTCTCGCCCCACGTTGAAAAGTCCATGCTGCGCATGCGCGAAACGGAGTGAAAGAAACGTTATTGCAACTGCGACTGCCCCCGCGTTTTGGGCTGGCCTTCTTATCAAGT |
| *dps Act5C_up_comm2* | ATATGGTAAACTTGGCTGCGGAATGGAGAAATGTCGCTGATAAGGAGTTGAGCGCAGCATGAGCGTGAGTGACGTAGCCGGCTGGAAAGCGGGCTTTATAAAACGGCAAGCAACGACCACGAATCAGTTTTTTTTTCGACGCAGCGCCTGCCGGACGTGAGCGTGCGGTTGAGCAAGATTCAGAACGATTATTCTCGTACCAACAAGAAGATACTGATACAGTGA |
| *dps CG12730* | TCTGGGCTCTCTCACTCAACGCTCCCCCACTCGGGGCTCTCTCTCTCTCGCTCTCGCTCACAATTGTAGTTTTTGTTATGGCATTTTTTCCGACGTCGCGTCGTCCAGCACACAAAATCGTTCTGAGTGGCGTTCAGTGGCGTTCTGTGCGTGCCCGTGAGCGCGCAATGTGAAAATATTTTCGCCGGGCTGTCGTCGTTGAAACCTTATCGAGTTGCTATTTCGTTTCGTTGTCGATTTTGTGCCTTTGCTGGAATGTCCATTATGTCCGCCAGCCAGGACTACGATCGGAAGTATGTGCGCAG |
| *dps comm2* | AGCAAGAGAGCGGAATGGAAATTGGAAGCCAACCGCTCTCAACTTCTCTCTTGACTGGAATTCCAGCGAGCGCTAGCTTGGCCGGCACTGCTGCTCTGTTTGAACGGCGCACGAATCAGTTTTTTTTTCGACGCAGCGCCTGCCGGACGTGAGCGTGCGGTTGAGCAAGATTCAGAACGATTATTCTCGTACCAACAAGAAGATACTGATACAGTGA |
| *dps comm2_TATA_insert* | AGCAAGAGAGCGGAATGGAAATTGGAAGCCAACCGCTCTCAACTTCTCTCTTGACTGGAATTCCAGCGAGCGCTAGCTTGGCCGGCTATAAAACTCTGTTTGAACGGCGCACGAATCAGTTTTTTTTTCGACGCAGCGCCTGCCGGACGTGAGCGTGCGGTTGAGCAAGATTCAGAACGATTATTCTCGTACCAACAAGAAGATACTGATACAGTGA |
| *dps dve* | CCACATGTTGCTATCCATTTGTGTGCGTGCCTCGGTGGCCGTTTCTGTATCTGCATCTGTGTGTCGGCGAGAATGTTTATGTGTGTATCCGTGTGTCATGCCTTTGCATGGGGGCTCTCGCAGCTGAGTTTCGGTTTCGGTTTCAGTATCTTTTCAAACATGCTCGAAGAAGGTTTGTTACTCTGCTCTCTCTTGCATCTCGAGTTTTCGGGTTCTCGGCTTCTTGTGTTTTGTGTGTTCCGCGCCGGTTTCCGTTCTAGTATCTGTGTGTGTTTCTGTTTGGCCGTATTTATGCTTGATGGCCAGCAATCGGATTATGTT |
| *dps dve_Act5C_inr* | CCACATGTTGCTATCCATTTGTGTGCGTGCCTCGGTGGCCGTTTCTGTATCTGCATCTGTGTGTCGGCGAGAATGTTTATGTGTGTATCCGTGTGTCATGCCTTTGCATGGGGGCTCTCGCAGCTGAGTTTCGGTTTAGTTTTCATATCATTATCAAACATGCTCGAAGAAGGTTTGTTACTCTGCTCTCTCTTGCATCTCGAGTTTTCGGGTTCTCGGCTTCTTGTGTTTTGTGTGTTCCGCGCCGGTTTCCGTTCTAGTATCTGTGTGTGTTTCTGTTTGGCCGTATTTATGCTTGATGGCCAGCAATCGGATTATGTT |
| *dps dve_inr_GtoA* | CCACATGTTGCTATCCATTTGTGTGCGTGCCTCGGTGGCCGTTTCTGTATCTGCATCTGTGTGTCGGCGAGAATGTTTATGTGTGTATCCGTGTGTCATGCCTTTGCATGGGGGCTCTCGCAGCTGAGTTTCGGTTTCGGTTTCAATATCTTTTCAAACATGCTCGAAGAAGGTTTGTTACTCTGCTCTCTCTTGCATCTCGAGTTTTCGGGTTCTCGGCTTCTTGTGTTTTGTGTGTTCCGCGCCGGTTTCCGTTCTAGTATCTGTGTGTGTTTCTGTTTGGCCGTATTTATGCTTGATGGCCAGCAATCGGATTATGTT |
| *dps dve_inr_GtoT* | CCACATGTTGCTATCCATTTGTGTGCGTGCCTCGGTGGCCGTTTCTGTATCTGCATCTGTGTGTCGGCGAGAATGTTTATGTGTGTATCCGTGTGTCATGCCTTTGCATGGGGGCTCTCGCAGCTGAGTTTCGGTTTCGGTTTCATTATCTTTTCAAACATGCTCGAAGAAGGTTTGTTACTCTGCTCTCTCTTGCATCTCGAGTTTTCGGGTTCTCGGCTTCTTGTGTTTTGTGTGTTCCGCGCCGGTTTCCGTTCTAGTATCTGTGTGTGTTTCTGTTTGGCCGTATTTATGCTTGATGGCCAGCAATCGGATTATGTT |
| *dps pepck* | CAACGGTGACCAATCAGCGCTCTCGCTCGCAAGCGCACCGATCGTGCACGATCGCGGAGCCGAACGCGCCTGTTCTAGGCGCATCGACGCCGAGGCACCCGCGGTAGCGTATAAAAGGCCGTGCGGCCTGGCATCTGGCATCAGTTACTCGTGTCCGGAGTAAATGGAAAGATCAAGTGAAAAGTCTACTTTCAGTGGTCTGAGCATCAAGCTGATATTTGACAAAAAGCTGTGGAAGAACATATGAAAGCGGGAGTCGCAC |
| *dps pepck_dve_down* | CAACGGTGACCAATCAGCGCTCTCGCTCGCAAGCGCACCGATCGTGCACGATCGCGGAGCCGAACGCGCCTGTTCTAGGCGCATCGACGCCGAGGCACCCGCGGTAGCGTATAAAAGGCCGTGCGGCCTGGCATCTGGCATCAGTTACTCAAACATGCTCGAAGAAGGTTTGTTACTCTGCTCTCTCTTGCATCTCGAGTTTTCGGGTTCTCGGCTTCTTGTGTTTTGTGTGTTCCGCGCCGGTTTCCGTTCTAGTATCTGTGTGTGTTTCTGTTTGGCCGTATTTATGCTTGATGGCCAGCAATCGGATTATGTT |
| *dps pepck_pk_down* | CAACGGTGACCAATCAGCGCTCTCGCTCGCAAGCGCACCGATCGTGCACGATCGCGGAGCCGAACGCGCCTGTTCTAGGCGCATCGACGCCGAGGCACCCGCGGTAGCGTATAAAAGGCCGTGCGGCCTGGCATCTGGCATCAGTTACTCAGACACGATCACGCGCGCGCACTCTCGCCCCACGTTGAAAAGTCCATGCTGCGCATGCGCGAAACGGAGTGAAAGAAACGTTATTGCAACTGCGACTGCCCCCGCGTTTTGGGCTGGCCTTCTTATCAAGT |
| *dps pino* | AATATCACTTTTGCGAGCTCGCTCTCTCGCTGTCTCTCTCTCCCACTGCTGTTTGCTGTGCTCTTCGTTTACGTACTCTCTCAGAGCCCGGCTATAAAAACGATGCGAACTGTGGCCAGCGCGTTCATTCGATTCCATCCATTCGCTTCGGTCGTGCATGCCACTTGGTCTTTGGCTTTCCAGAAAAAATCAATTCCGTTTGTCTCAACGGAAGCCCAACTATAAAAGTGCGCTGTATCAAAACAATCGTTATTATTTGGATATCAACCCGATGAACACTGAGCAATACGTTTACACGAGCTAACTGTCTCCATCGCGTGTGAAAA |
| *dps pk* | CTGTGGCTGTGTTCCCAGTATCAATCGTTCTGCGATTACCAGTATTTCCAGTGCCGAAAGAGCGAGCGCTCGGGAGAGCGACCCCCAGTGGGAGAGCGTGCTAGTCGGCGCACGACTCCCCGATTGGAGTTCGTCGTTGGGGCCGATCGCAGCGCGCCGTCAGTCTGCTTTCAGACACGATCACGCGCGCGCACTCTCGCCCCACGTTGAAAAGTCCATGCTGCGCATGCGCGAAACGGAGTGAAAGAAACGTTATTGCAACTGCGACTGCCCCCGCGTTTTGGGCTGGCCTTCTTATCAAGT |
| *dps pk_tata_insert* | CTGTGGCTGTGTTCCCAGTATCAATCGTTCTGCGATTACCAGTATTTCCAGTGCCGAAAGAGCGAGCGCTCGGGAGAGCGACCCCCAGTGGGAGAGCGTGCTAGTCGGCGCACGACTCCCCGATTGGAGTTATAAAATGGGGCCGATCGCAGCGCGCCGTCAGTCTGCTTTCAGACACGATCACGCGCGCGCACTCTCGCCCCACGTTGAAAAGTCCATGCTGCGCATGCGCGAAACGGAGTGAAAGAAACGTTATTGCAACTGCGACTGCCCCCGCGTTTTGGGCTGGCCTTCTTATCAAGT |
| *dps pk_inr_GtoT* | CTGTGGCTGTGTTCCCAGTATCAATCGTTCTGCGATTACCAGTATTTCCAGTGCCGAAAGAGCGAGCGCTCGGGAGAGCGACCCCCAGTGGGAGAGCGTGCTAGTCGGCGCACGACTCCCCGATTGGAGTTCGTCGTTGGGGCCGATCGCAGCGCGCCGTCATTCTGCTTTCAGACACGATCACGCGCGCGCACTCTCGCCCCACGTTGAAAAGTCCATGCTGCGCATGCGCGAAACGGAGTGAAAGAAACGTTATTGCAACTGCGACTGCCCCCGCGTTTTGGGCTGGCCTTCTTATCAAGT |
| *dps pxb* | GCCGAAACACGATTGAGCACGATGCGACAGACGTGCACGAGCATGTCTGCCTGCCTGGCTTGCCTGCCTGCCGAGCAACATGTTCGCAGGCAGCGCCAGCAACATTTGCAGGCGGGCGGTTTTTGAAAACACGGCCTTGCCGCCCGAATGAATGTATCTCACAAGCCGGCAACCAGTTACCGTTCAGTTTTTCCATCAACGTCACCGACATCGGACGTGGTGCGTCTCGCGTTATTTTCCATCTCAACGATCTGAGTTTTTGTCCAACGGAATACGAAA |
| *dps RpL13A* | TCTTTCTTTCAATCTCGTTAAAGTGGACATATAAATACTGAAAAATACCAGCGGTATATTTTGAATATGAGACGGTATATTTCAGACGGTCGGACGGTATATTTGATCATTTATCGACCAAAGTCACTCTGTCGAGCTGTCAACGCAGTTTCCGCTTTCTTTCCGTGCGGTTCTTAAACATGACTGGTTTAACGAATAGGGTAAGTGCCAACAAAAATGGAAAATAAACTTATTTAATGTGACGTTATTACCGAACAAATGTATGCAAAGTGTTGAGTGCCAT |
| *dps RpL13A 2kb* | ATTTTGGTACCTTCGTTGTTCGACATCAACAGCAACCGCCTCAGCTTCTAGGTGAGTCAATTGCAATTGAAATTACATTTTTGGAATCTAAATCTAATTTACCTGCTTTTCTTTCTGTTGTACTGTAGCTGCTTTCCTGGGAGTGTTCCCCCAAAATCCACGACACAAAGTCACCGAGTTGAGATAGCCATGGATAATTTAAGTTCTCTTAAAAAAAAATAGCATAATAAGAATCAAAATAAAATCGCAATGTCATGGCATTAAAAGTAACACCATTAATAAGAACGCCTTTTTCTAGTTTAGACAGAGCAGCGCTCTATCGCTGTTTCTTTCTTTCAATCTCGTTAAAGTGGACATATAAATACTGAAAAATACCAGCGGTATATTTTGAATATGAGACGGTATATTTCAGACGGTCGGACGGTATATTTGATCATTTATCGACCAAAGTCACTCTGTCGAGCTGTCAACGCAGTTTCCGCTTTCTTTCCGTGCGGTTCTTAAACATGACTGGTTTAACGAATAGGGTAAGTGCCAACAAAAATGGAAAATAAACTTATTTAATGTGACGTTATTACCGAACAAATGTATGCAAAGTGTTGAGTGCCATGTGGAGTTGTGGTGACAGTGATAAAATGCTGCGAATAGCACCGAAAATCTTGTGTTTTTAGTGGCGAAACCATTTCGCACAGTACACGTGCCCATGCTAAAAGTGGGAGCCTGGCATATGTGAAAATGATAAAATGGTGCGCACAATGAGCATTGGGCTGAGTGAAATGTGTGCACGACAGTTTTTTGACGAACGTGAACTGGCGGGAAATGCTCTGCATAAATTTGTTTATACGGCGTTCACAAAACATAACCTCACATTTCTGACATTGTTTTTGTGCTCCCCTTGCAGACCGTTGTTATTGATGGTCGCGGCCATTTGCTTGGCCGTCTGGCTTCCGTCGTCGCCAAGTACCTGTTGCAGGGCGGCAAGGTGGCCGTCGTTCGCTGCGAGGAGCTGAACCTCTCCGGCCACTTCTACAGAAACAAAATCAAGTTCTTGGCCTACTTGCGCAAGCGTTGCAACGTCAACCCAGCTCGTGGTCCCTTCCACTTCCGTGCCCCGTCCAGGATCTTCTACAAGGCGGTCAGAGGTATGTACAGGCCTAAGGCGAGTGGAGTTCAGCGAATCTGTGATGTTTTCAATCATATTACCTACATTATTTGAATGAGAAACCATGTAAACAAAACATAACTGAGTGAAGCTGGTCAACACATTCCTGCCTATCCCCTCTGAAATCATGTATTTACTGCCTTTTATTTTCTTTCAGGTAAGCTCGAGTTTCAGAGGTCCTCCGAACTAACTTTCGTGATGATTATTATCTTACCTACATTATTTGAACGAGAAGTCGTGTAACCAACCTCAAACTGATCCGCTCATGAGATCATCAAGCCTGTTTATGCTATAGTGTCTTTACTAATGCAAAGGTTTTTCTCCCTCGCAGGTATGATCCCACACAAGACTAAGCGTGGCCAGGCTGCACTCGCTCGTCTGCGTGTGTTCGATGGCATCCCATCGCCCTACGACAAGCGTCGCCGCGTCGTTGTCCCCATCGCTATGCGTGTGTTGACACTGCGCTCCGACCGCAAGTACTGCCAGGTCGGCCGTCTGTCGCACGAGGTTGGCTGGCACTACCAGGATGTGATCAAGAGCCTGGAGCGTAAGCGCAAGGCCAAGTTGCGTGTCACCCTCAAGCACAACCGCGAGCTGAAGAAGCTGACTGTTCAGGCACGTGAAAACATTGCCAAGGCTGCTGAGCCCTTCAACAAAATCATCAAATCCTATGGCTACGAGGTTTAAGGACAACAAAAACACCGGCAGATGCGTAGCAGTTTTTTAAAGGGAATAAAACAACATCTAAGTTTAAAAAACAATCGTCTTGTG |
| SCP | TTGTAAAACGACGGCCAGTGAATTCGAGCTCGGTACCCGGGGATCCTCTAGAGTACTTATATAAGGGGGTGGGGGCGCGTTCGTCCTCAGTCGCGATCGAACACTCGAGCCGAGCAGACGTGCCTACGGACCGCTGCAGGCATGCAAGCTTGGCGTAATC |
| SCP_inr_GtoT | TTGTAAAACGACGGCCAGTGAATTCGAGCTCGGTACCCGGGGATCCTCTAGAGTACTTATATAAGGGGGTGGGGGCGCGTTCGTCCTCATTCGCGATCGAACACTCGAGCCGAGCAGACGTGCCTACGGACCGCTGCAGGCATGCAAGCTTGGCGTAATC |

**Table S8. Reporter backbone sequence and promoter sequences.**
