# Supplementary material for: Reporter-ChIP-nexus reveals strong contribution of the Drosophila initiator sequence to RNA polymerase pausing
Source: eLife. 2019 Apr 25;8:e41461. doi: 10.7554/eLife.41461 (PMC6483594; doi:10.7554/eLife.41461)
Supplement: Transparent reporting form [file elife-41461-transrepform.docx]

***eLife’s* transparent reporting form**

We encourage authors to provide detailed information *within their submission* to facilitate the interpretation and replication of experiments. Authors can upload supporting documentation to indicate the use of appropriate reporting guidelines for health-related research (see [EQUATOR Network](http://www.equator-network.org/%20)), life science research (see the [BioSharing Information Resource](https://biosharing.org/)), or the [ARRIVE guidelines](http://www.plosbiology.org/article/info:doi/10.1371/journal.pbio.1000412) for reporting work involving animal research. Where applicable, authors should refer to any relevant reporting standards documents in this form.

If you have any questions, please consult our Journal Policies and/or contact us: [editorial@elifesciences.org](mailto:editorial@elifesciences.org).

**Sample-size estimation**

- You should state whether an appropriate sample size was computed when the study was being designed
- You should state the statistical method of sample size computation and any required assumptions
- If no explicit power analysis was used, you should describe how you decided what sample (replicate) size (number) to use

Please outline where this information can be found within the submission (e.g., sections or figure legends), or explain why this information doesn’t apply to your submission:

10^7^cell were used for each ChIP-nexus experiment, this information can be found within section “ChIP-nexus”.

For quantifying the changes in paused Pol II half-life after promoter sequence mutagenesis, two biological replicates were performed using 10^7^ transfected cells each. This information can be found within figure 3-C legend and section “Normalization of Pol II signal on the plasmid”.

For quantifying the changes in gene expression after promoter sequence mutagenesis, cDNA and DNA were prepared from two to three biological replicates using 5×10^5^ transfected cells each. Two technical replicates were performed during qPCR. This information can be found within figure 7-figure supplement 3 legend and section “reporter expression quantification”.

For analyzing the differences in initiator sequences between TATA and stably paused promoters, 132 promoters were included in each group. This information can be found within figure 6-A legend and section “Sequence analysis of TATA promoters and stably paused promoters”.

For analyzing the relationship between paused Pol II half-life and core promoter elements, promoters with paused Pol II half-life info were separated based on the presence of core promoter elements following either a mutually exclusive model or a non-mutually exclusive model. The number of promoters in each group is shown in figure 4-A, figure 7-A, figure 4-figure supplement 1 and figure 7-figure supplement 1. The detailed separation methods are described under section “Correlation between paused Pol II half-lives and core promoter elements”

**Replicates**

- You should report how often each experiment was performed
- You should include a definition of biological versus technical replication
- The data obtained should be provided and sufficient information should be provided to indicate the number of independent biological and/or technical replicates
- If you encountered any outliers, you should describe how these were handled
- Criteria for exclusion/inclusion of data should be clearly stated
- High-throughput sequence data should be uploaded before submission, with a private link for reviewers provided (these are available from both GEO and ArrayExpress)

Please outline where this information can be found within the submission (e.g., sections or figure legends), or explain why this information doesn’t apply to your submission:

For quantifying the changes in paused Pol II half-life after promoter sequence mutagenesis, two biological replicates were performed using 10^7^ transfected cells each. This information can be found within figure 3-C legend and section “Normalization of Pol II signal on the plasmid”.

For quantifying the changes in gene expression after promoter sequence mutagenesis, cDNA and DNA were prepared from two to three biological replicates using 5×10^5^ transfected cells each. Two technical replicates were performed during qPCR. This information can be found within figure 7-figure supplement 3 legend and section “reporter expression quantification”.

**Statistical reporting**

- Statistical analysis methods should be described and justified
- Raw data should be presented in figures whenever informative to do so (typically when N per group is less than 10)
- For each experiment, you should identify the statistical tests used, exact values of N, definitions of center, methods of multiple test correction, and dispersion and precision measures (e.g., mean, median, SD, SEM, confidence intervals; and, for the major substantive results, a measure of effect size (e.g., Pearson's r, Cohen's d)
- Report exact p-values wherever possible alongside the summary statistics and 95% confidence intervals. These should be reported for all key questions and not only when the p-value is less than 0.05.

Please outline where this information can be found within the submission (e.g., sections or figure legends), or explain why this information doesn’t apply to your submission:

Statistical methods used for plotting error bar and analyzing the correlation between Pol II profile on the plasmid and that at the endogenous locus are stated in figure legends and table legends.

Wilcoxon rank test was used for testing the significance of differences in paused Pol II half-life between promoters with different combinations of core promoter elements. This information can be found within the figure legends of figure 4-A, 7-A and table S4

(For large datasets, or papers with a very large number of statistical tests, you may upload a single table file with tests, Ns, etc., with reference to sections in the manuscript.)

**Group allocation**

- Indicate how samples were allocated into experimental groups (in the case of clinical studies, please specify allocation to treatment method); if randomization was used, please also state if restricted randomization was applied
- Indicate if masking was used during group allocation, data collection and/or data analysis

Please outline where this information can be found within the submission (e.g., sections or figure legends), or explain why this information doesn’t apply to your submission:

For analyzing the differences in initiator sequences between TATA and stably paused promoters, TATA promoters and stably paused promoters were identified using methods under section “Sequence analysis of TATA promoters and stably paused promoters”.

For analyzing the relationship between paused Pol II half-life and core promoter elements, promoters with paused Pol II half-life info were separated based on the presence of core promoter elements following either a mutually exclusive model or a non-mutually exclusive model. The number of promoters in each group is shown in figure 4-A, figure 7-A, figure 4-figure supplement 1 and figure 7-figure supplement 1. The detailed separation methods are described under section “Correlation between paused Pol II half-lives and core promoter elements”

**Additional data files (“source data”)**

- We encourage you to upload relevant additional data files, such as numerical data that are represented as a graph in a figure, or as a summary table
- Where provided, these should be in the most useful format, and they can be uploaded as “Source data” files linked to a main figure or table
- Include model definition files including the full list of parameters used
- Include code used for data analysis (e.g., R, MatLab)
- Avoid stating that data files are “available upon request”

Please indicate the figures or tables for which source data files have been provided:

Raw sequencing data and analysis code availability is stated within section “Data and software availability”
